# Irradiated obese adipocytes are prone to oxidative stress, inflammation, senescence, and fibrosis

**DOI:** 10.1038/s41598-025-23533-7

**Published:** 2025-11-13

**Authors:** Simran Takkar, Elizabeth A. Kosmacek, Joshua A. McDowell, Kia T. Liermann-Wooldrik, Arpita Chatterjee, Rebecca E. Oberley-Deegan

**Affiliations:** Department of Biochemistry and Molecular Biology, University of Nebraska Medical Center, Omaha, NE 68198 USA

**Keywords:** Obese adipocytes, Radiation, ROS, Inflammation, Senescence, Fibrosis, Endocrine system and metabolic diseases, Senescence

## Abstract

Cancer patients with obesity who undergo radiotherapy are more likely to suffer from side effects caused by the radiation-induced damage to normal tissues adjacent to the tumor. Exposure of these normal tissues during radiation treatment results in post-irradiation related chronic damage leading to fibrosis. Obese adipose tissue is associated with oxidative stress promoting oxidative damage, senescence, inflammation, and fibrosis. However, the effect that radiation has on obese adipose tissue during the radiation treatment is unknown. In the present work, to study the obese conditions we utilized 3T3-L1 adipocytes exposed to fatty acid cocktail for an in vitro model and C57/BL/6 mice exposed to high fat diet (HFD) for an in vivo obese model. We established a clinically relevant dosing model of pelvic radiation of 7.5 Gy, 1.2 Gy/min, 160 KV X-ray over 5 days to analyze the effect of radiation in the context of obesity for both models. We found that obese and irradiated obese adipose released free fatty acid and glycerol (1–2 fold compared to control) and this was accompanied by elevated (2–3 fold compared to control) oxidative damage in vitro and in vivo. Irradiated HFD adipose displayed high oxidative stress, senescence, inflammation, and fibrosis as compared to obesity alone, which may induce cancer progression and recurrence.

**Supplementary Information:**

The online version contains supplementary material available at 10.1038/s41598-025-23533-7.

## Introduction

 The incidence of obesity is rising worldwide and is considered a global epidemic^1^. Various factors, such as lifestyle, genetics, and epigenetics play major roles in obesity and weight gain^2^. Obesity is a strong risk factor for type 2 diabetes mellitus, cancer, cardiovascular disease, and nonalcoholic fatty liver disease^3^. White adipose tissue (WAT) is a complex organ and plays a primary role in energy homeostasis. Adipocytes act as an energy reservoir that sense energy demands and secrete paracrine factors such as cytokines and chemokines to regulate other metabolic organs^4,5^. In obesity, WAT becomes dysfunctional and expands resulting in deleterious effects including fibrosis, inflammation, mitochondrial dysfunction, and altered adipokine secretion, each of the factors are potential therapeutic targets of the treatment of obesity^6,7^.

Radiation is an important therapy used to treat various cancer types and stages, such as head and neck, brain, breast, rectal, and prostate cancer^8–12^. Radiation leads to acute damage of biomolecules promoting water hydrolysis and the formation of double-stranded DNA breaks. This leads to the generation of reactive hydroxyl radicals and other reactive oxygen species (ROS). The accumulation of ROS promotes oxidative stress, inflammation, and fibrosis through the activation of NF-ĸB or TGF-β signaling^13–15^. Radiotherapy is an effective anti-cancer therapy as it allows for maximal dose targeting to the tumor and its impact is more deleterious to fast dividing cells, like cancer^16,17^. Unfortunately, radiation therapy, including in the pelvic region, causes damage to the surrounding normal organs and tissues^18^. The damage to normal tissues during radiation results in chronic inflammation and fibrosis.

Patients with obesity that undergo radiotherapy, especially in pelvic cancers such as prostate cancer, exhibit higher metastatic potential and tumor recurrence^19,20^. Adipose tissue expands in obesity, surrounding the organs in the pelvic region making this tumor microenvironment unique^21^. However, the radiation treatment plans for patients with obesity do not spare adipose tissue. Although a few studies have investigated the impact of radiation on normal and healthy patients^22,23^, there are no studies that have investigated the molecular and physiological changes in adipose tissue during radiotherapy in the context of obesity.

Therefore, our study addresses this gap of knowledge by reporting the different molecular effects of radiation on obese adipose tissue. In this manuscript, we have established in vitro and in vivo obesity models and have reported the effect of radiation on the physiological and molecular changes including metabolic dysfunction, oxidative stress, senescence, inflammation, and fibrosis of obese adipose tissues.

## Results

### Establishment of in vitro and in vivo obesity models

**Fig. 1:**
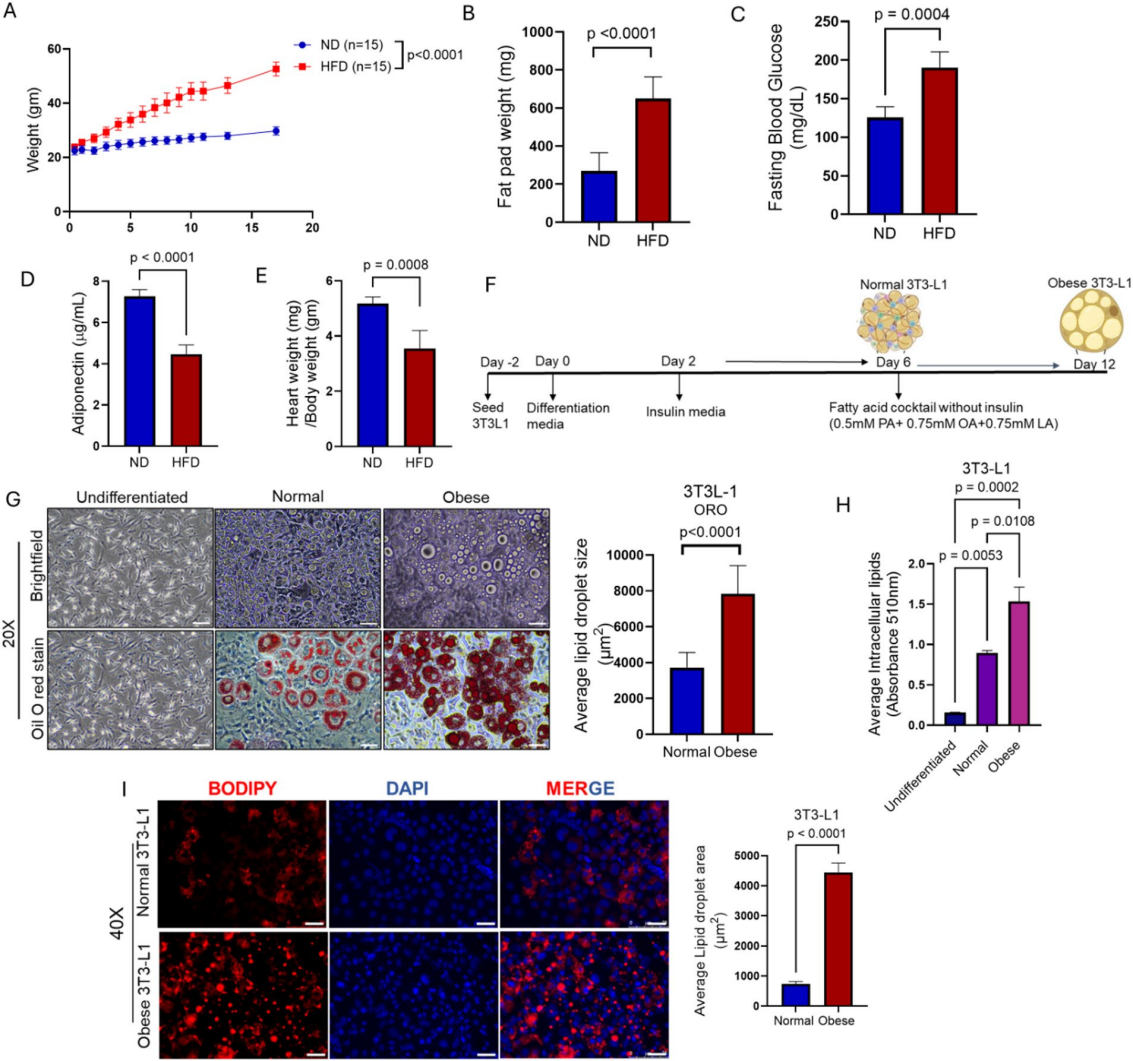
Establishing in vitro and in vivo obesity models.** (A)** Body weight of mice fed with HFD and ND for 16 weeks. **(B)** Weight of WAT excised from HFD and ND mice. **(C)** Measurement of fasting blood glucose levels of HFD and ND mice. **(D)** Estimation of adiponectin from the serum of HFD and ND mice. **(E)** Weight of heart excised from the HFD and ND mice. (*n* = 15 mice/group) **(F)** Schematic representation of in vitro obesity model. **(G)** Left, representative brightfield and Oil red O stained images of undifferentiated, normal, and obese 3T3-L1 adipocytes. Right, quantification of lipid droplet size from Oil red O images (4–5 images/biological replicate, *n* = 3) Image was taken at 200X magnification. **(H)** Quantitative analysis of Oil red O-stained lipid droplets at 510 nm. Statistical significance was analyzed by one-way ANOVA followed by post hoc Tukey’s test for multiple comparisons. **(I)** Left, representative images stained with BODIPY C_16_ that stains neutral lipids. Right, quantification of the neutral lipids stained with BODIPY C_16_ (4–5 images/biological replicate, *n* = 3) Image was taken at 200x magnification. The white bar represents 100 μm. Statistical significance was calculated using Student’s t-test and results are expressed as mean ± SEM. *n* = 3 independent experiments.

To establish a diet-induced obesity model, C57/BL/6 mice were fed a high fat diet (HFD) or normal diet (ND) for 16 weeks. As expected, mice fed with HFD developed the hallmarks of obesity phenotype including elevated body weight, increased fat pad mass, hyperglycemia, and reduced adiponectin levels (Fig. 1A, B, C, D). Moreover, obesity is one of the major risk factors for cardiac disease due to reduced heart weight and heart rate^24^. Similarly, in our diet-induced obesity mouse model the heart weight was significantly decreased compared to ND mice (Fig. 1E). These results suggest the establishment of an in vivo obesity model. However, to study the obesity associated conditions in vitro, we utilized the combination of fatty acid cocktail including palmitic acid, oleic acid, and linolenic acid, as described previously^25^. Briefly, 3T3-L1 cells were differentiated for 48 h and then incubated in insulin media for 4 consecutive days. This was followed by the addition of the fatty acid cocktail on day 6 through day 12, as described^25^(Fig. 1F). On day 6 and day 12, the lipid accumulation profiles were analyzed using a brightfield microscope followed by Oil Red O stain to visualize the lipids. We found that 3T3-L1 cells accumulated more lipids in the presence of the fatty acid cocktail as compared to normal and undifferentiated 3T3-L1 cells, which mimics adipocyte hypertrophy found in obese conditions (Fig. 1G). Similar results were observed in 3T3-L1-MBX adipocytes (Supplementary Fig. 1). Quantitative analysis of the accumulated Oil Red O stain indicated a 2-fold increase in lipid accumulation in obese 3T3-L1 cells compared to undifferentiated and normal matured 3T3-L1 cells (Fig. 1H). Furthermore, we also performed a BODIPY C_16_ assay, fluorescent lipophilic stain to observe the lipid droplets. Our results revealed larger lipid droplets in the presence of the fatty acid cocktail (Fig. 1I). These outcomes are in line with the experimental and epidemiological evidence in other obesity studies^25–28^. Taken together, these results demonstrate the establishment of in vitro and in vivo obesity models to study the effect of radiation on obesity associated conditions.

### Radiation induces metabolic changes in obese conditions

**Fig. 2:**
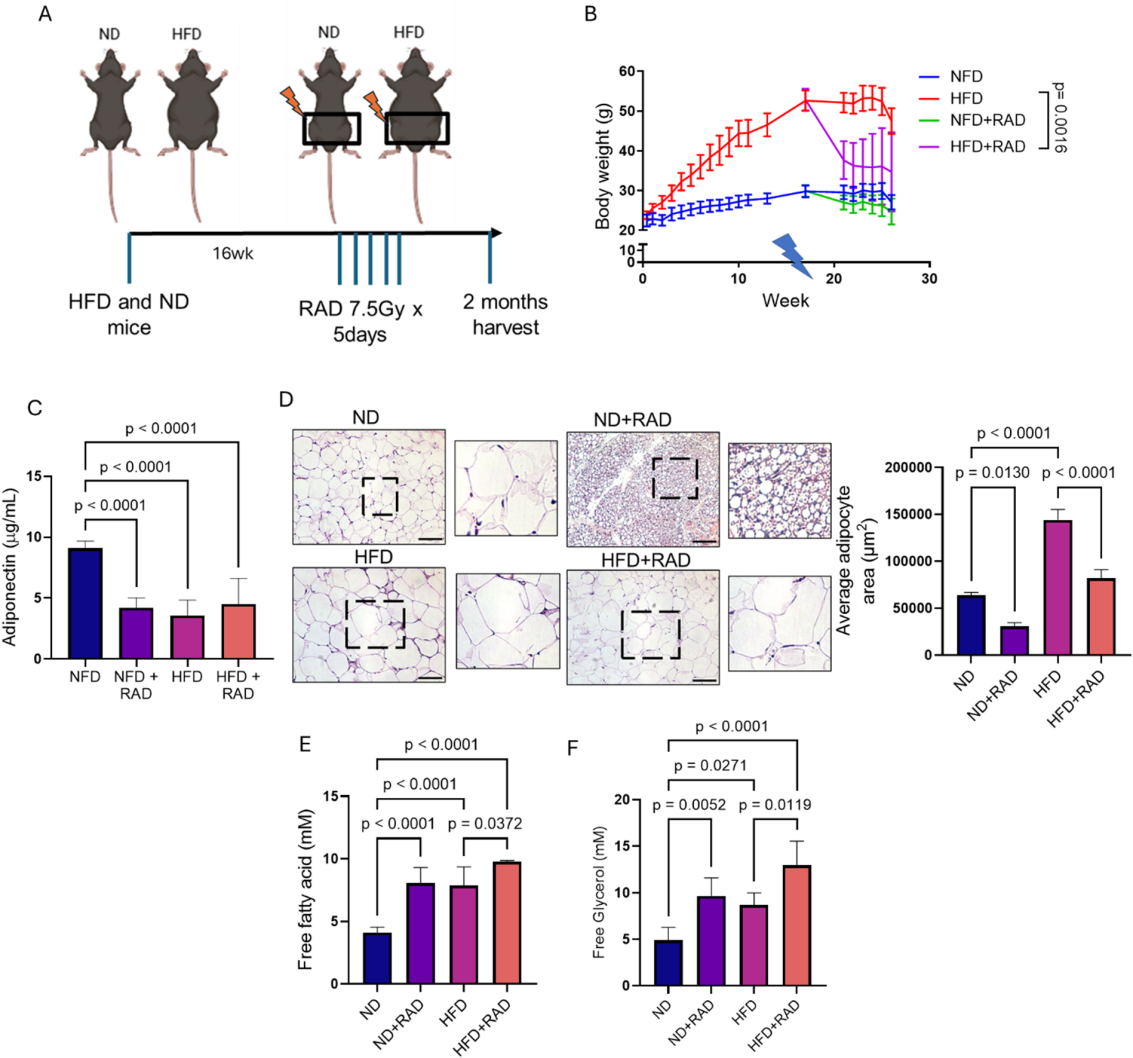
Radiation enhances metabolic alterations in obese conditions.** (A)** Schematic representation of HFD and ND mice receiving 7.5 Gy of radiation for five consecutive days pelvically before harvest at 2 months post-radiation. **(B)** Weight of mice fed with HFD and ND after receiving 7.5 Gy of pelvic radiation (ND, *n* = 7, ND + RAD, *n* = 8, HFD, *n* = 7, HFD + RAD, *n* = 8). **(C)** Adiponectin concentrations estimated from the serum of ND, HFD, irradiated ND and HFD mice (*n* = 5 mice/group). **(D)** Left, representative H&E images of adipose tissue of unirradiated and irradiated HFD and ND. Black box is the is the area that is magnified an additional 10X and shown at the immediate right. Right, quantification of adipose area (Images were traced and size quantified (4–5 images/mouse, 5 mice/group). **(E**,** F)** Free fatty acid and free glycerol concentrations from the serum of unirradiated and irradiated HFD and ND mice (5 mice/group). The data are given as the mean ± SEM (4–5 images were randomly collected and analyzed per mouse, 5 mice/group). Statistical significance was calculated using one-way ANOVA followed by post hoc Tukey’s test for multiple comparisons. The black bar represents 100 μm.

To elucidate the role of radiation in obese conditions. We radiated the pelvis (2 × 2 cm^2^) of C57/BL/6 that were on ND or HFD diet for 16 weeks with 7.5 Gy of radiation for 5 consecutive days at 1.2 Gy/min using a RadSource 2000 X-Ray Box Irradiator (Fig. 2A). We chose to investigate gonadal fat pads as they resemble human visceral adipose tissue that have been implicated in obesity related pathogenesis^29^. Next, the weight of the mice was determined two months post-radiation. Interestingly, we found significantly reduced body weight of HFD that received radiation compared to their unirradiated controls, respectively (Fig. 2B). Next, we determined the adiponectin levels post-radiation from the collected serum of HFD and ND mice (Fig. 2C). Circulating adiponectin levels were significantly lower in the serum of HFD mice compared to ND mice as observed in Fig. 1D and reported previously (Fig. 2C)^30,31^. In addition, the adiponectin levels decreased post-radiation in mice that were fed ND, but no significant difference in the adiponectin levels was observed in obese mice post-radiation as compared to HFD alone (Fig. 2C). The H&E staining of the WAT demonstrated that adipose size expanded in the obese (HFD) conditions as compared to control adipose tissue (ND). However, the size of the adipocytes was reduced in ND and HFD mice at two months post-radiation (Fig. 2D). Physiological changes in the adipose tissue can alter metabolism and release of free fatty acids, chemokines, and cytokines^6^. Therefore, we performed an ELISA for free fatty acid and free glycerol in the serum of ND and HFD mice that received pelvic radiation. As expected, elevated levels of free fatty acid and free glycerol were detected in obese conditions as compared to control (Fig. 2E, F). Interestingly, the levels of free fatty acid and free glycerol increased post-radiation in the serum of both ND and HFD mice and these levels were similar between irradiated ND and HFD mice alone (Fig. 2E, F). Irradiated HFD mice had significantly more free fatty acid and free glycerol as compared to other groups. Overall, the data indicates physiological changes in the adipose tissue of obese mice alone and irradiated HFD and ND mice.

### Radiation induces oxidative damage in obese conditions in vitro and in vivo

**Fig. 3:**
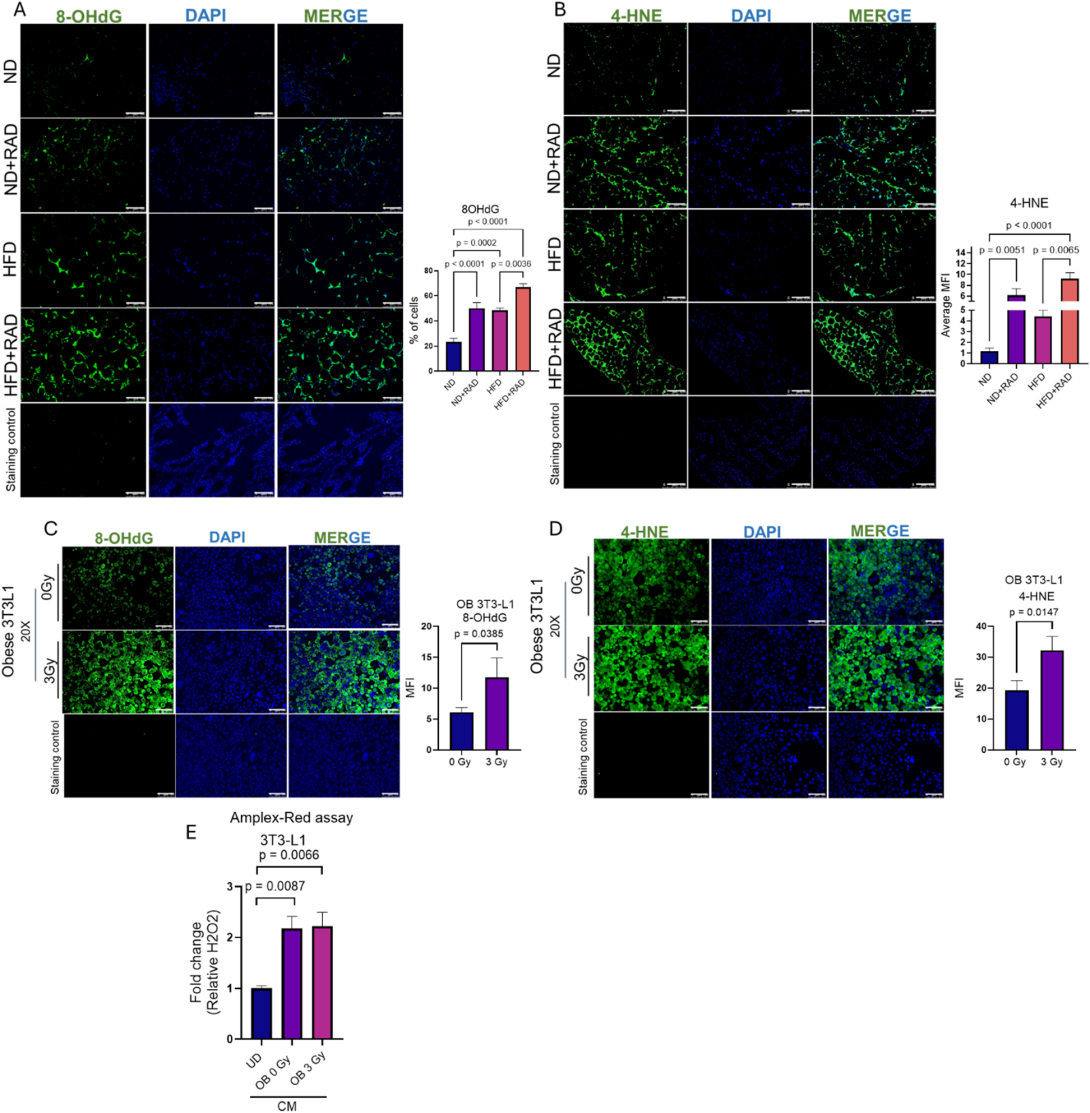
Radiation increases the accumulation of oxidative stress in obese adipocytes in vivo and in vitro. To determine radiation-induced oxidative stress in the adipose tissue, HFD and ND fed mice were irradiated at 7.5 Gy for five consecutive days or 0 Gy. Adipose tissue was excised 2 months later. **(A)** Left, adipose tissue sections were stained with 8-OHdG, a DNA/RNA oxidation indicator. Right, quantification of 8-OHdG staining. **(B)** Left, adipose tissue stained with 4-HNE, lipid peroxidation. Right, quantification of 4-HNE staining. A minimum of five images were captured and averaged for quantification per mouse, 5 mice/group. The data represents the mean of ± SEM (*n* = 5 mice/group). No primary antibody was used as a negative staining control. A one-way ANOVA with post-hoc Tukey test was used to calculate significance. **(C)** Left, representative images of obese 3T3-L1 stained with 8-OHdG in the presence of 3 Gy of radiation after 48 h or 0 Gy. Right, quantification of 8-OHdG staining in unirradiated and irradiated obese 3T3-L1 cells. **(D)** Left, immunofluorescence images of 4-HNE in obese and irradiated obese 3T3-L1 adipocytes at 3 Gy of radiation after 48 h or 0 Gy radiation. Right, quantification of 4-HNE staining in obese adipocytes at 3 Gy–0 Gy. 4–5 images/biological samples were averaged for quantification, *n* = 3. Image was taken at 200X magnification. Statistical significance was assessed by Student’s t-test. **(E)** Hydrogen peroxide levels from the conditioned media of obese and obese 3T3-L1 cells 48 h post-radiation. UD = Undifferentiated, CM = Condition media. The white bar represents 100 μm. All in vitro experiments were repeated *n* ≥ 3. For all the experiments the data represents the mean ± SEM.

Previously published studies have established that obesity is associated with oxidative stress^6,32,33^. Exposure to radiation induces reactive oxygen species (ROS) and accumulation of oxidative stress^34^. Therefore, we stained adipose tissue of obese and normal diet mice with 8-hydroxy-2-deoxyguanosine (8-OHdG) to measure DNA/RNA oxidation and 4-hydroxynoneonal (4-HNE) to investigate lipid peroxidation. As expected, we found increased 8-OHdG and 4-HNE staining in HFD as compared to ND adipose tissue sections (Fig. 3A, B). We also observed that radiation caused increased 4-HNE and 8-OHdG staining in HFD mice post-radiation as compared to HFD alone (Fig. 3A, B). We found that the staining of 4-HNE and 8-OHdG were similar between ND irradiated adipose tissue and HFD adipose tissue and that HFD irradiated mice had the highest markers of oxidative stress (Fig. 3A, B). Next, we validated our findings at the in vitro level utilizing obese 3T3-L1 adipocytes. We assessed oxidative stress levels 48 h after exposure to 0 Gy of radiation or 3 fractions of 3 Gy of radiation for 3 days in the presence of fatty acid conditions to mimic the obese conditions during radiation treatment. Endogenous 8-OHdG staining was observed in the obese 3T3-L1 adipocytes using immunofluorescence suggesting obesity itself is associated with DNA damage (Fig. 3C). Further, we observed a significant increase in the 8-OHdG staining in the obese 3T3-L1 adipocytes post-radiation, similar to the in vivo findings (Fig. 3C). Endogenous expression of 4-HNE in obese 3T3-L1 adipocytes was observed and elevated post-radiation (Fig. 3D). Next, we performed an Amplex-Red assay to determine the accumulation of H_2_O_2_ from the conditioned media collected from obese and obese radiated cells 48 h post-radiation. Before radiation exposure on day 12, the media on the obese and obese radiated 3T3-L1 cells was changed to remove the free fatty acids from the media as the free fatty acid can promote ROS. We found elevated levels of H_2_O_2_ in the media of obese and obese radiated adipocytes as compared to the undifferentiated 3T3-L1 cells, indicating that H_2_O_2_ may be a ROS involved in enhancing radiation induced damage markers (Fig. 3E).

### Radiation elevates senescence in obese adipocytes

**Fig. 4:**
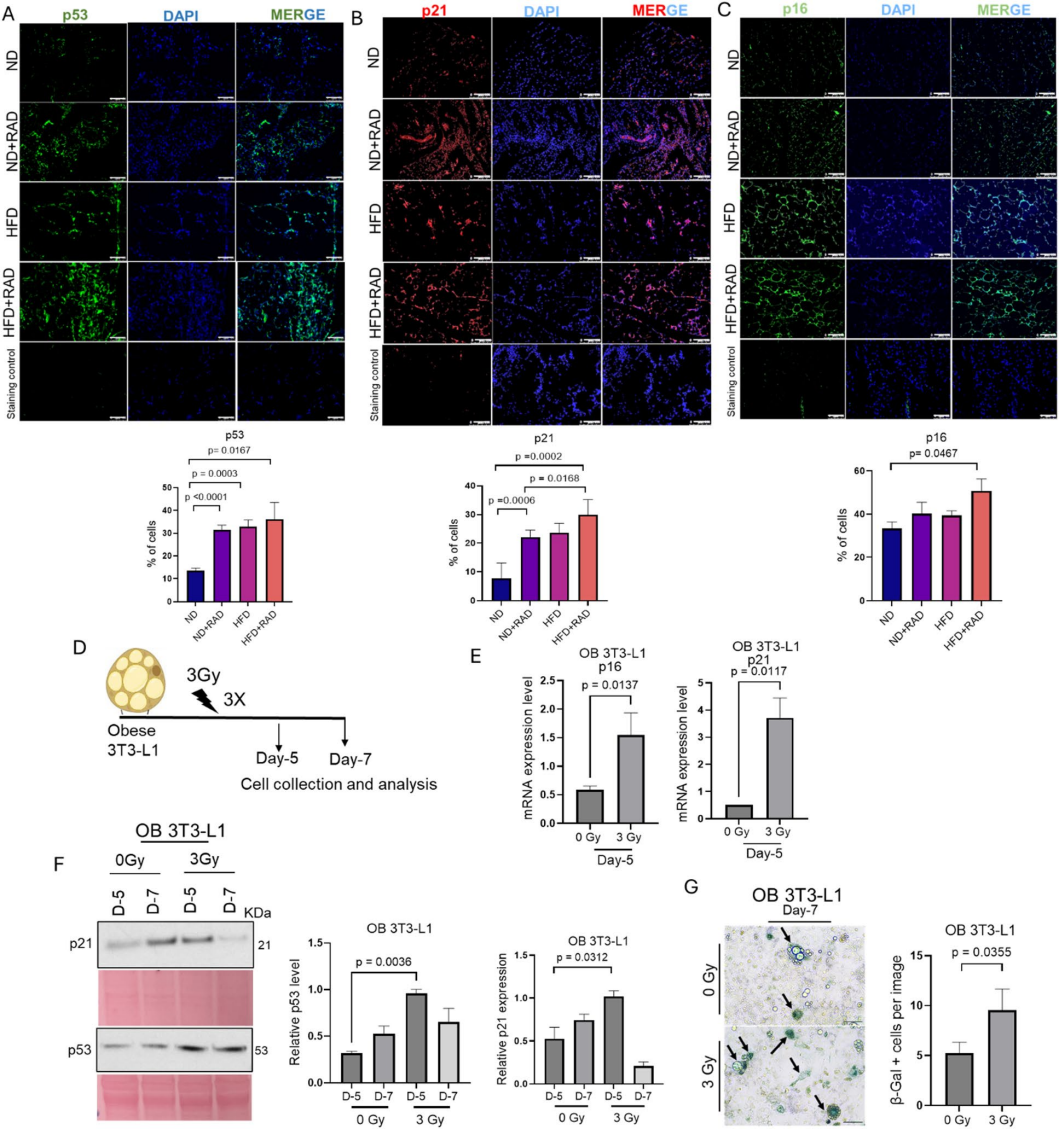
Radiation elevates the senescence markers in obese conditions in vivo and in vitro. **(A**,** B**,** C)** Top, representative images and quantification of adipose tissue sections stained with p53 (p53 = green, DAPI = blue), p21 (p21 = red, DAPI = blue), p16 (p16 = green, DAPI = blue); cellular senescence marker in the HFD and ND fed mice alone and 2 months post-radiation. Bottom, quantification of p53, p21, and p16 staining in adipose tissue. A minimum of 5 images were taken per mouse and averaged for quantification and the data represents the mean ± SEM (*n* = 5 mice/group). Images were taken at 200X. No primary antibody was used as a staining control. Statistical analysis was determined using one-way ANOVA followed by post-hoc Tukey test for multiple comparisons. **(D)** Schematic methodology for the collection of obese 3T3-L1 cells after 3 days of 3 Gy radiation or 0 Gy control in the presence of fatty acids to mimic the obese conditions. **(E)** RT-qPCR analysis of p16 and p21 relative mRNA in obese 3T3-L1 adipocytes at day 5 post-radiation or unirradiated. **(F)** Left, representative western blot of p21 and p53. Right, quantification of p21 and p53 in obese 3T3-L1 adipocytes at day 5 and 7 post-radiation or 0 Gy. Ponceau was used as a loading control. p53 and p21 expression was normalized to their respective Ponceau images. Supplementary Fig.S2 presents the original membranes of western blot. **(G)** Left, representative images of β-galactosidase staining in obese 3T3-L1 cells seven days post-radiation or non-radiation. Right, quantification of β-galactosidase staining. Statistical analysis was determined using either one-way ANOVA followed by post-hoc Tukey test or a Student’s t-test. All the experiments were repeated three times and data represents the mean ± SEM, *n* = 3 biological independent samples. The white and black bars represens 100 μm.

Oxidative stress can cause DNA damage, which induces senescence stress via the activation of the p53-p21 pathway and the p16 axis^35,36^. Cellular senescence is a multifaceted state in which cells stop dividing but remain metabolically active^37^. Previous studies have shown that in obesity, adipose tissue is susceptible to cellular senescence and genome instability^38,39^. However, the effect of radiation on senescence in the context of obesity has not been studied. Therefore, we performed immunohistochemistry for senescence markers p53, p21, and p16 on the adipose tissue from HFD and ND mice two months post-radiation. Interestingly, we found significantly elevated levels of p53, and p21 in the HFD adipose tissue as compared to ND, p16 levels trended to be elevated in HFD as compared to ND but was not significant (Fig. 4A, B,C). Radiation caused a slight increase in p53, p21, and p16 staining in HFD adipose tissue post-radiation, but was not significant (Fig. 4A, B,C). Interestingly, HFD and irradiated ND adipose tissue had similar levels of senescent markers (Fig. 4A, B,C). Subsequently, to determine whether obese 3T3-L1 adipocytes have a cellular senescence phenotype post-radiation, we utilized our obese 3T3-L1 established model. We radiated obese 3T3-L1 adipocytes at 3 Gy for 3 days or 0 Gy for 3 days in obese conditions and then the cells were collected at 5 and 7 days post-radiation for western blot and RT-qPCR analysis (Fig. 4D). Using RT-qPCR analysis, we found significantly elevated levels of p16 and p21 at day 5 in the irradiated obese 3T3-L1 as compared to non-irradiated obese adipocytes (Fig. 4E). Next, we performed western blots and found the endogenous protein expression of p21 and p53 in obese adipocytes were elevated post-radiation at day 5, validating our RT-qPCR data (Fig. 4F). However, we were not able to detect any bands for p16 (data not shown). At day 7 post-radiation, p53 levels were still elevated but p21 protein had decreased (Fig. 4F). The state of senescence is characterized by the upregulation of senescence-associated beta-galactosidase (β-gal) activity^37,40^. Seven days post-radiation, we observed enhanced accumulation of β-gal positive senescent cells compared to non-radiated obese adipocytes (Fig. 4G). Taken together, our findings demonstrate that radiation elevates the accumulation of p16, p21, and p53 in the adipose tissue of HFD and HFD irradiated mice in vivo and activation of the p21-p53 pathway in vitro.

### Radiation promotes chronic inflammation in obesity

**Fig. 5:**
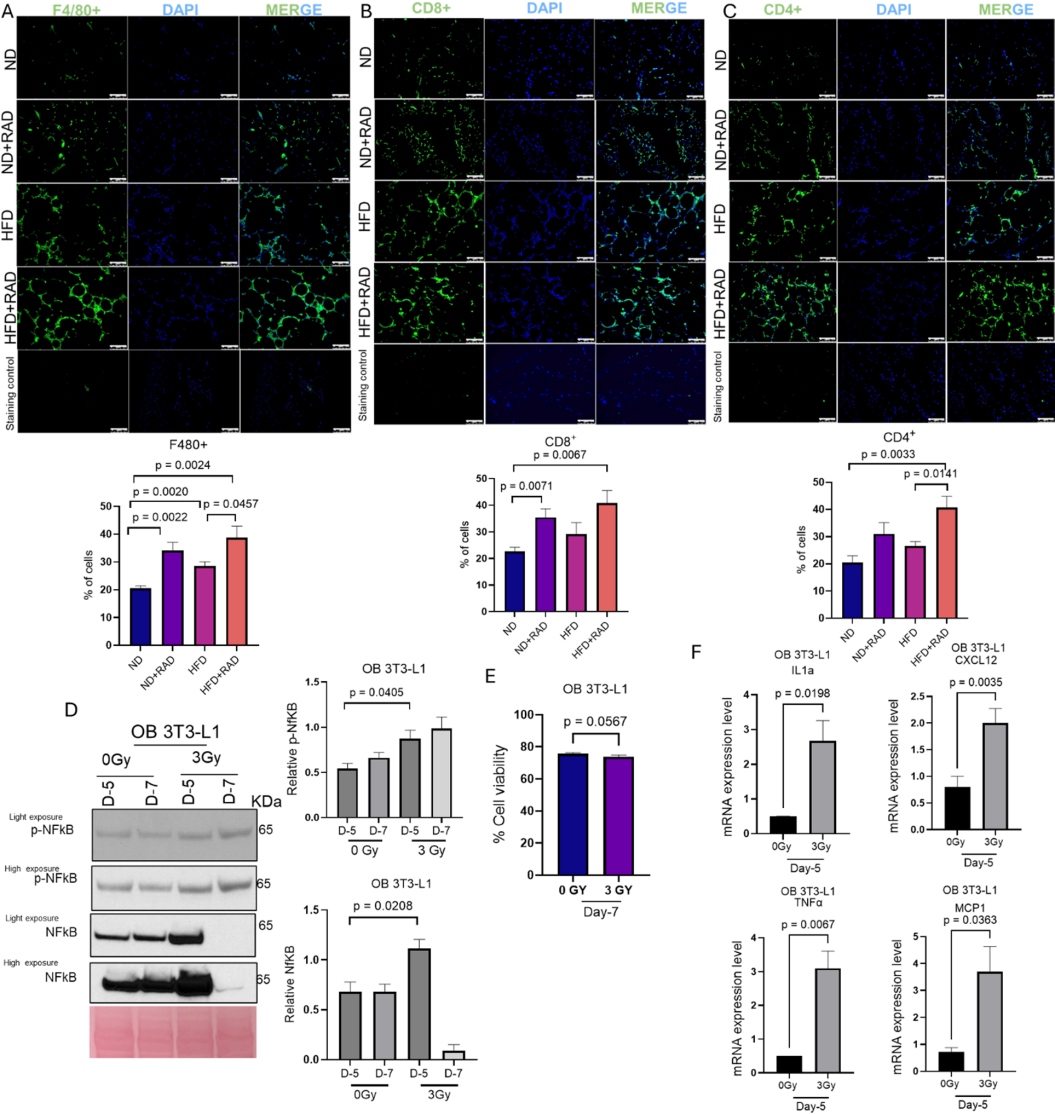
Radiation leads to elevated immune infiltration in vivo and promotes pro-inflammatory signaling in in vitro models of obesity.** (A**,** B**,** C)** Top, representative images of adipose tissue sections of HFD and ND mice alone at two months post-radiation stained with F4/80, CD8+, and CD4+. Bottom, quantification of F4/80, CD8+, and CD4 + staining. A minimum of 5 images were captured per mouse and averaged for the quantification, *n* = 5 mice/group. The data represents the mean of ± SEM. No primary antibody was used as a staining control. Images were taken at 200X. The white bar represents 100 μm. **(D)** Left, representative western blot of phosphorylated or total NF-ĸB and Right, quantification of p-NF-ĸB and total NF-ĸB at day 5 and day 7 post- radiation. Ponceau was used as a loading control. Phosphorylated and total NF-ĸB expression were normalized to Ponceau (loading control). Supplementary Fig.S3 presents the original membranes of western blot. **(E)** Viability of obese and irradiated obese 3T3-L1 cells on day 7. **(F)** RT-qPCR analysis of pro-inflammatory cytokines in obese 3T3-L1 adipocytes at day 5 post- radiation or no-radiation. All in vitro experiments were repeated three times, *n* = 3, and data represent the mean ± SEM. Statistical difference was calculated using either one-way ANOVA followed by post-hoc Tukey’s test or a Student’s t-test.

We showed that radiation promotes metabolic dysfunction and oxidative stress leading to the accumulation of senescent cells in obese conditions. The activation of cellular senescence contributes to the induction of chronic inflammation^41^. Moreover, obesity and weight gain lead to phenotypic alteration of adipocytes (as observed in H&E) and characterized by infiltration of immune cells that produce crown-like structures^6,42^. Therefore, we immunostained the adipose tissue sections from irradiated and unirradiated HFD and ND mice for total macrophages (F4/80^+^). There were significant elevated levels of total macrophages in the crown like structures of adipose tissue of HFD mice as compared to ND (Fig. 5A). We also observed that radiation led to increased infiltration of macrophages in HFD post-radiation compared to HFD fed mice alone (Fig. 5A). Consistent to the other abovementioned results, HFD adipose tissue and irradiated ND adipose tissue were similar two months post-radiation (Fig. 5A). Adipose tissue macrophages present antigens to initiate the recruitment of other immune cells, and they release cytokines to stimulate an inflammatory signaling cascade^6,43^. Therefore, we stained the unirradiated and irradiated adipose tissue sections of HFD and ND for CD8^+^ and CD4^+^ T cells. Elevated levels of CD8^+^ and CD4^+^ immune cells were found in HFD adipose tissue as compared to ND (Fig. 5B, C). A significant increase in the CD4^+^ cells was seen in HFD mice post-radiation as compared to unirradiated HFD mice (Fig. 5B, C). The HFD adipose tissue and ND radiated adipose tissue sections had similar levels of CD4^+^ and CD8^+^ cells (Fig. 5B, C). Next, we wanted to validate the activation of inflammation-related signaling at the in vitro level. Therefore, we irradiated obese 3T3-L1 adipocytes with 3 Gy for 3 days or 0 Gy for 3 days in the presence of fatty acids and performed a western blot to visualize the activation of the NF-ĸB transcription factor. ^44,45^. The western blot analysis revealed the endogenous expression of phosphorylated and total NF-ĸB in obese 3T3-L1 cells was elevated 5 days post-radiation (Fig. 5D). Because we observed the reduction in the NF-ĸB and p21 (Fig. 4F) expression at day 7 post-radiation, we checked the viability of cells. We did not find any significant changes in the viability of obese 3T3-L1 cells 7 days post-radiation (Fig. 5E). The activation of NF-ĸB leads to the production of pro-inflammatory chemokines, cytokines, and adhesion molecules^44^. Therefore, we analyzed the release of several pro-inflammatory cytokines in unirradiated and irradiated obese 3T3-L1 conditions by RT-qPCR. We found the upregulation of several pro-inflammatory cytokines, such as, interleukin1a (IL1a), C-X-C motif chemokine 12 (CXCL12), tumor necrosis factor (TNF-α), and C-CL2 chemokine (also known as monocyte chemoattractant protein-1, MCP1) at 5 days post-radiation (Fig. 5F). Altogether, these results suggest that radiation elevates the infiltration of immune cells in the adipose tissue of HFD mice. Moreover, radiation leads to the phosphorylation of NF-ĸB and the release of pro-inflammatory cytokines in the obese 3T3-L1 adipocytes.

### Radiation induces pro-fibrotic signaling in obese adipocytes

**Fig. 6:**
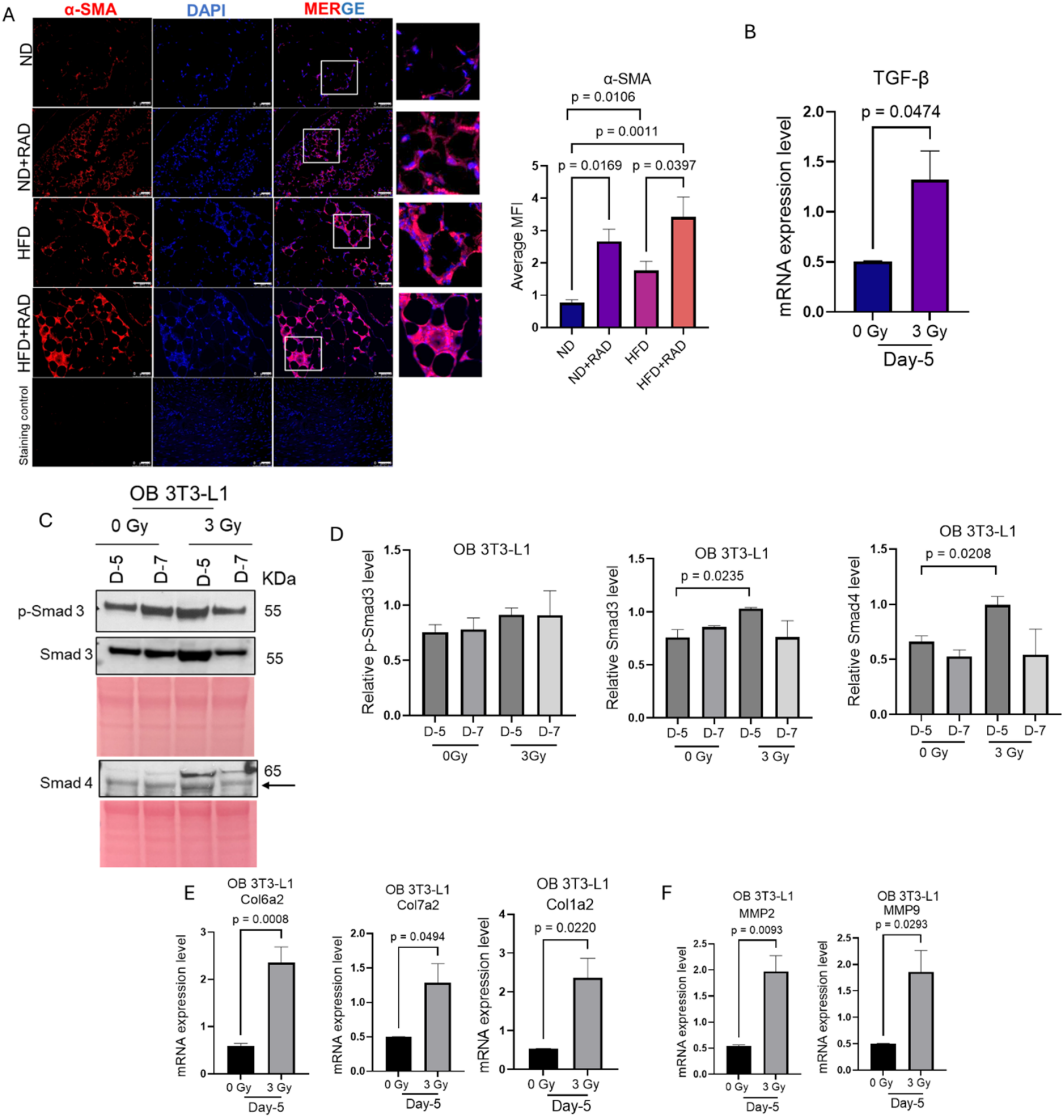
Radiation elevates pro-fibrotic pathways in obese conditions.** (A)** Left, representative images of α-SMA staining in the adipose tissue sections of HFD and ND mice at two months post-radiation. The white box is the area that is magnified an additional 10X and shown at the immediate right. Right, quantification of α-SMA staining. 4–5 images were taken per mouse and averaged for quantification, *n* = 5 mice/group. A one-way ANOVA followed by post-hoc Tukey test was used to determine statistical significance. The data represents the mean ± SEM (*n* = 5 mice/group). Images were captured at 200X. No primary antibody was used as a negative staining control. (**B)** RT-qPCR analysis of TGF-β in obese 3T3-L1 adipocytes at day 5 post-radiation or 0 Gy. (**C)** Representative western blot images of p-Smad3, total Smad3, and Smad4 at day 5 and day 7 post-radiation. Ponceau was used as a loading control. The expression of p-Smad3, Smad3, and Smad4 was normalized to their respective ponceau (loading control). Supplementary Fig.S4 presents the original membranes of the western blot. **(E**) RT-qPCR analysis of Col6a2, Col1a2, and Col7a2 in obese 3T3-L1 adipocytes at day 5 post-radiation or no radiation. **(F**) RT-qPCR analysis of MMP2 and MMP9 in obese 3T3-L1 adipocytes at day 5 post-radiation or no-radiation. Statistical analysis was calculated using either one-way ANOVA post-hoc Tukey test or Student’s t-test. All in vitro experiments were repeated three times *n* = 3 biological independent samples. The white bar represents 100 μm.

Previous studies have shown that inflammation promotes fibrosis^45,46^ Therefore, we investigated the effect of radiation in promoting profibrotic features in the obese environment at the in vitro and in vivo levels. First, we performed immunofluorescence for α-SMA, a pro-fibrotic marker of myofibroblasts, in the adipose tissue of HFD and ND alone and in the presence of radiation. We observed elevated α-SMA staining in HFD adipose tissue as compared to ND (Fig. 6A). We also noticed a significant increase in the α-SMA staining of irradiated HFD adipose tissue as compared to unirradiated HFD alone (Fig. 6A). As found with other endpoints, HFD and irradiated ND adipose tissue had similar levels of α-SMA staining (Fig. 6A). Next, to determine the fibrotic phenotype pathways in *vitro*, we utilized irradiated and unirradiated obese 3T3-L1 cells and performed mRNA expression for TGF-β, a known inducer of fibrosis^47–49^. Our results revealed elevated gene expression of TGF-β at 5 days post-radiation (Fig. 6B). We then checked the TGF-β-Smad signaling by western blot. We observed a significant increase in the levels of total Smad 3 and Smad 4 at 5 days post-radiation in the obese 3T3-L1 adipocytes (Fig. 6C, D). A trend of increased expression of phosphorylated Smad 3 was observed but the change was not significant among the groups (Fig. 6D). The induction of TGF-β-Smad signaling leads to the upregulation of collagens and matrix metalloproteases (MMPs) that promote extracellular matrix (ECM) deposition and degradation^47,50,51^. Therefore, we checked the gene expression of different collagens and MMPs in our irradiated and unirradiated obese 3T3-L1 adipocytes by RT-qPCR. We found radiation caused significant increases in the gene expression of Col6a2, Col7a2, and Col1a2 at day 5 post-radiation (Fig. 6E). Additionally, we also observed the elevation in the MMP2 and MMP9 expression in the obese adipocytes 5 days post-radiation (Fig. 6F). Overall, this data suggests that obesity itself is associated with fibrosis and radiation further promotes this phenotype both in vivo and in vitro.

## Discussion

Obesity is not only associated with increased risk of cancer but also contributes to cancer mortality and cancer recurrence^52,53^. Radiation therapy remains the first line of treatment for many cancer patients. However, normal tissues surrounding the tumor are not spared during radiation exposure. In addition, there are no studies looking at the effects of radiation in obese adipocytes. Radiotherapy causes the formation of ROS in the normal adjacent tissues leading to inflammation, fibrosis, and decreased organ function^54^. Therefore, it is very important to understand how adipose tissue is affected during radiation treatment in the context of obesity.

This is the first study to investigate radiation toxicity in the context of obesity using in vivo and in vitro obesity models. In the present study, to mimic the obese conditions we have used an in vivo diet-induced model by exposing C57/BL/6 mice to 60% high fat diet and an in vitro model by adding a fatty acid cocktail to adipocytes. Further, to investigate the impact of radiation on obesity, we created the clinically relevant in vivo model by exposing HFD and ND mice to 7.5 Gy of radiation for five consecutive days to the pelvic region. We then created an in vitro model of obese patients undergoing radiotherapy, where obese 3T3-L1 cells were exposed to 3 Gy of radiation for three consecutive days in the presence of fatty acids cocktail.

Excessive accumulation of fat exposed to radiation causes oxidative stress promoting cytokine production, DNA/RNA damage, and lipid peroxidation^55–57^. Our results also demonstrate the increased accumulation of extracellular H_2_O_2_ in the obese and obese radiated 3T3-L1 cells, which could be a possible reason for the elevated oxidative stress markers observed in the unirradiated and irradiated obese adipose *in vitro and in vivo*.

A pro-oxidatively stressed environment promotes the cellular senescence that triggers the release of an aberrant secretome including profibrotic and proinflammatory factors^58–60^. Our study is the first report to investigate the effect of radiation on senescence associated with obesity. We have successfully shown that irradiated HFD adipose tissue had more accumulation of senescence markers compared to all the groups and that HFD mice and ND irradiated mice showed similar phenotypic effects of senescence. In vitro, we were able to establish the upregulation of p21/p53 axis at 5 days post-radiation. Subsequently, we saw a decrease in the p21 expression but not in p53 at day 7 post-radiation, indicating that the expression of p21 is likely independent of p53, which has been shown in other studies^61–63^.

The obese adipose tissue is associated with chronic inflammation that leads to reorganization of extracellular matrix, fibrosis, and altered angiogenesis^64–68^. In our study, we have highlighted the elevated infiltration of macrophages (F4/80^+^) and other immune cells, such as, CD8^+^ and CD4^+^ in the HFD irradiated adipose tissue compared to all the groups. In vitro, we reported the activation of the NF-ĸB pathway and the release of proinflammatory cytokines in the presence of radiation. We did observe a decrease in the total NF-ĸB levels 7 days post-radiation, but the phosphorylated form was still upregulated. This data indicates that NF-ĸB levels may fluctuate with radiation exposure.

Fibrosis is characterized by the differentiation and proliferation of cells into myofibroblasts causing the contraction and deposition of ECM^69–71^. TGF-β induces pro-fibrotic gene expression by binding to the TGF-β receptor, resulting in the phosphorylation of Smad2 or Smad3. The phosphorylated Smad then binds to Smad4 and translocates to the nucleus and turns on the expression of collagens, and MMPs leading to ECM remodeling^49^. Our results reveal that irradiated HFD mice had the highest marker of myofibroblasts compared to all the groups. In vitro, our data expanded the induction of autocrine TGF-β fibrotic signaling pathway in the obese 3T3-L1 adipocytes post-radiation through the Smad signaling pathway.

Some limitations of the study are that we only used 3T3 cells for in vitro experiments. Future studies should also use obese WAT adipocytes from HFD and irradiated HFD mice. Secondly, the experiments are performed in male C57/BL/6 mice, these findings should be validated in female mice and no potential differences between mouse and human adipose radiation responses were evaluated. Third, we only focused on WAT for this study other fat tissues may respond differently to radiation, and this will be a future direction. Fourth metabolic differences, such as hyperglycemia or altered FFA/free glycerol associated with obesity, could influence the observed responses to radiation. Although we focused on tissue-specific effects, these systemic metabolic changes may act as confounding factors. Future studies incorporating comprehensive metabolic profiling will be important to delineate the direct effects of radiation from those influenced by underlying metabolic dysfunction. Fifth, future studies are required to elucidate the effect of obesity and radiation on a tumor as this study only focuses on the effects of normal tissues. Sixth, our study focuses solely on tissue-specific effects at two months post-radiation. Including a time-course analysis of changes in the tissue would provide a more comprehensive understanding of the temporal dynamics of radiation-induced effects on obese tissues. Lastly, no cause and effect were established between oxidative stress, inflammation, senescence, and fibrosis as this was an observational study. It is important to determine the relationship between oxidative stress, senescence, and inflammation contributing to fibrosis.

In conclusion, we discovered that obese WAT has increased oxidative stress, metabolic dysfunction, inflammation, and fibrosis compared to normal WAT. Furthermore, radiation exposure significantly contributed to these phenotypes in obesity. In addition, radiation damages adipose tissue regardless of dietary conditions. Additionally, irradiated ND adipose had significantly elevated oxidative stress, and inflammatory phenotypes as observed in our previously published study^72^. An interesting finding from the current study is that irradiated ND mice and HFD alone mice had similar adipose damage suggesting irradiated normal fat resembles obese fat. It is well documented that obesity promotes pancreatic, prostate, and breast cancer and worse overall survival in these patients^43,73,74^. Moreover, radiation increases metastasis and cancer recurrence in the lung, breast, and prostate cancer patients^75–78^. Therefore, future studies are required to elucidate the effect of obese and irradiated obese adipose conditions in promoting the tumor microenvironment, and tumor progression that could result in cancer recurrence.

## Materials and methods

### Cell culture

Mouse embryonic stem cells, 3T3-L1 were purchased from ATCC (CL-173). Cells were maintained in 10% fetal bovine serum (FBS) and 1% penicillin/streptomycin containing DMEM media. 3T3-L1 adipocytes were differentiated with differentiation media containing 10 µg/ml insulin, 1 µM dexamethasone, 0.5 mM IBMX in DMEM media for 48 h. The 3T3-L1 cells that were not exposed to differentiation media are stated as undifferentiated 3T3-L1.

### Fatty acids treatment

To mimic the obese conditions in vitro, the combination of fatty acid cocktail was used containing saturated, mono-unsaturated, and polyunsaturated fatty acids as reported previously^25,79^. Briefly, after differentiating cells in the differentiating media for 48 h, cells were incubated with 10 µg/ml insulin containing DMEM media for four days. At this point, the cells were treated with the combination of fatty acid cocktail containing 0.5 mM palmitic acid (Cat# P5585, Millipore Sigma), 0.75 mM oleic acid (Cat# O3008, Millipore Sigma), and 0.75 mM linolenic acid (Cat# L9530, Millipore Sigma or BML-FA014-1000, Enzo) in no insulin DMEM media. The cocktail media was changed after every two days. After 12 days, cells exhibited an obese phenotype which contained large lipid droplets and elevated lipid accumulation as visualized by bright field microscope and Oil Red O staining.

### Diet induced obesity mouse model

4-5week-old C57/BL/6 male mice were obtained from Jackson laboratories. The mice were housed at the University of Nebraska Medical Center (UNMC) in accordance with the Guide for Care and Use of Laboratory Animals by National Institute of Health. Mice were fed and watered *ad libitum* and kept on 12-hour light/dark cycle. All animal experiments were approved by UNMC Institutional Animal Care and Use Committee (IUCAC; 20-019-03-FC) and the study was conducted in compliance with the ARRIVE guidelines. The studies also comply with the IUCN Policy Statement on Research Involving Species at Risk of Extinction and the Convention on the Trade in Endangered Species of Wild Fauna and Flora. 4–5 week old C57/BL/6 male mice were randomly separated within two groups and fed with a high fat diet [HFD 60% kcal lard fat, D12492i Research Diets; macronutrient composition: protein (Casein, Lactic Cystine L); carbohydrate (Lodex 10, sucrose fine granulated); fiber (Solka Floc FCC200); vitamin (Choline Bitartrate)]  or normal diet (ND 7012 Teklad, Inotiv) for 16 weeks. After 16 weeks, the body weight of mice, blood glucose level, heart weight, and fat pad weight were determined. To measure fasting blood glucose levels mice were fasted overnight. Next day, blood was drawn via the saphenous vein and the glucose was measured using a commercially available glucose meter (Kroger Health Pro meter). Animals were euthanized with CO_2_ inhalation followed by cervical dislocation.

### Radiation treatments

**Fig. 7:**
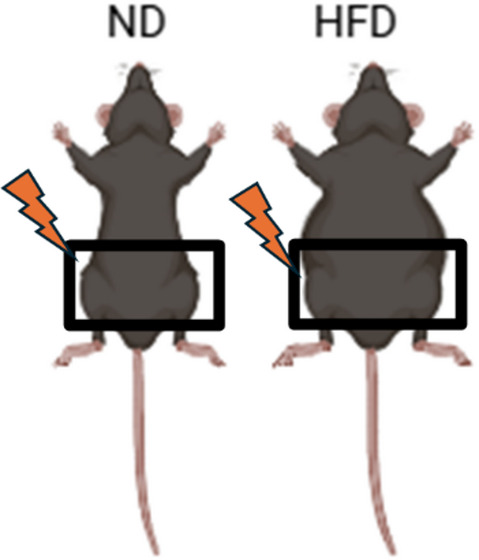
Schematic representation of the exact pelvic region of C57/BL/6 mice that received 7.5 Gy of radiation for 5 consecutive days.

The HFD and ND C57/BL/6 mice were randomized in four groups using a random number generator following the diet stabilization: HFD No-irradiation (HFD alone *n* = 7), HFD irradiation (HFD + RAD *n* = 8), ND No irradiation (ND *n* = 7), and ND irradiation (ND + RAD *n* = 8). The mice were anesthetized with a xylazine (11 mg/kg) and ketamine (80 mg/kg) solution intraperitoneally (ip). The upper body was lead-shielded exposing the pelvis to x-irradiation at 1.2 Gy/min. Both ND and HFD mice received 7.5 Gy for 5 consecutive days using a RadSource 2000 X-Ray Box Irradiator (Fig. 7). We collected adipose tissue at two months post-radiation from five mice in each of the four groups. Investigators were blinded during IHC staining, image capture and image analysis. For in vitro studies, the obese cells were radiated at 3 Gy for 3 consecutive days (fractionated exposure, 1.98 Gy/min, 160 KV) or sham (0 Gy) using a RadSource 2000 X-ray Box Irradiator in the presence of fatty acid cocktail and remained in fatty acid media until the samples were collected at day-5 and 7 post-radiation for analysis to mimic the obese patients undergoing radiation treatment. The RadSource 2000 X-ray Box Irradiator is calibrated yearly using an ion chamber. The dose of radiation applied and uniformity of the dose to the mice was verified with an ion chamber and radiographic film in phantoms. .

### Oil red O staining

4% paraformaldehyde was used to fix the undifferentiated, normal, and obese 3T3-L1 adipocytes for 10–15 min. Next, the cells were washed with PBS and then the Oil Red O stain (Cat# 01391, Sigma-Aldrich 3:2 ratio with DI water) was added so that the cells were completely covered for 5–10 min. The Oil Red O stain was removed with tap water. Next, brightfield images of Oil Red O stained lipid droplets were captured using an Olympus IX81 inverted microscope. The captured images were quantified using ImageJ software. For intracellular lipid measurement, the Oil Red O-stained cells were then incubated in 60% isopropanol for 2–5 min and the eluted Oil Red O stain was quantified using an Infinite M200 Pro plate reader (Tecan) at 510 nm.

### BODIPY

To detect the neutral lipids, 3T3-L1 adipocytes were stained with 10 µM BODIPY FL C16 (Cat# D3821, Invitrogen) for 2 h and then washed with PBS to remove excess stain. After 2 h, cells were cytospun on glass slides. Nuclei were stained with DAPI. Images were taken on a Leica DM4000 B LED microscope at 505/512 nm and quantified using ImageJ.

### Estimation of adipocyte size

Hematoxylin and eosin-stained tissues were used to measure and quantify adipocyte area at two months post-radiation. The brightfield images were captured on a Leica DM4000 B LED microscope. Adipocyte area was quantified using ImageJ by tracing the borders of the adipose cells.

### Free fatty acid and free glycerol assays

Two-months post-radiation, ND and HFD mice were euthanized and blood was collected. The collected blood was then centrifuged at 3000 x g for 10 min, and the serum was extracted and stored at −20 °C. Serum free glycerol kit (Cat#65337, Abcam) and free fatty acid kit (Cat#65341, Abcam) were measured as per the manufacturer’s protocol.

### Adiponectin assays

After euthanization, blood was collected from the thoracic cavity and centrifuged for 10 min at 3000 x g. After centrifugation, the serum was stored at −20 °C. An ELISA for adiponectin (Abcam 108785) was performed from the collected serum according to the manufacturers protocol as reported previously^80^.

### Amplex red assay

On day 12, the media of obese cells was changed from free fatty acid media to 0.2% FBS containing DMEM media. The media from obese and irradiated obese adipocytes were collected at 48 h post-radiation to measure the extracellular H_2_O_2_ using Amplex Red H_2_O_2_ assay (Cat# A36006, Invitrogen). Absorbance was read from 570 to 590 nm.

### Cell viability

The cellular viability of obese and irradiated obese 3T3-L1 adipocytes was determined seven days post-radiation using trypan blue exclusion. Briefly, the medium of obese and irradiated obese adipocytes were collected and cells were trypsinzed, centrifuged and resuspended in 1 ml of fresh media. Cells were then mixed in 1:1 ratio with trypan blue and loaded on the hemocytometer. The live and dead cells were counted to calculate the percentage of viable cells.

### Quantitative RT-qPCR

Five days post-radiation, total RNA was isolated from obese 3T3-L1 adipocytes using an Qiagen RNA isolation kit. The purity of the isolated RNA was analyzed using Tecan Infinite 200Pro nanodrop. RNA (1µg) was utilized for cDNA synthesis by Applied Biosystems (Cat#4368814). SYBR Green Master Mix (Roche) was used for quantitative real-time PCR (RT-qPCR). The cycle conditions included 95 °C for 5 minutes, 30 seconds for 95 °C, 58 °C for 1 minute, and 30 seconds at 72 °C for 39 cycles. RPLPO (F-5’-GCAGGTGTTTGACAACGGCAG-3’, R-5’-GATGATGGAGTGTGGCACCGA-3’) was used to normalize expression levels, and the calculated data is presented as fold changes compared to control (unirradiated samples). We did not observe any changes in the stability of RLPLO under the radiation and treatment of fatty acids. The details of the mouse primer used in the study are p21 (F-5’-CCCGCCTTTTTCTTCTTAGC-3’; R-5’-TTCTCATGCCATTCCTTTCC-3’), p16 (F-5’-CCCAACGCCCCGAACT-3’; R-5’-GCAGAAGAGCTGCTACGTGAA-3’), IL1a (F-5’- GCAACGGGAAGATTCTGAAG-3’; R-5’ TGACAAACTTCTGCCTGACG-3’), TNF-α (F- 5’- CATCTTCTCAAAATTCGAGT-3’; R-5’ TGGGAGTAGACAAGGTACAA-3’), CXCL12 (F- 5′-GGACGCCAAGGTCGTCGCCGTG-3′; 5′- TTGCATCTCCCACGGATGTCAG-3′), and MCP1 (F-5’-GCTGGGATTCACCTCAAGAA-3’; R- 5’-AGGTGCCATCAGAGCAGTCT-3’).

### Western blotting

Obese 3T3-L1 adipocytes were irradiated with 3 Gy for 3 consecutive days or sham irradiated as a control. Unirradiated and radiated obese 3T3-L1 cells were collected at five and seven days post- radiation. The cells were lysed in lysis buffer containing 50 mM Tris- HCl, 1% NP-40, 120 mM NaCl, 5 mM EDTA, and complete protease inhibitor cocktail tablets (1 tablet/50 ml; Roche, cat # 11697498001) were added for the preparation of whole cell extract as described previously^81^. The protein was quantified using a BCA kit and samples (20–40 µg per well) were resolved on 4–12% Bis-Tris plus gels (Thermo Fisher Scientific, cat # NW04120BOX) followed by transfer to nitrocellulose membranes (Life Technologies, cat # IB23002). Membranes were then blocked for 1 h in non-fat dry milk in Tris buffer saline containing 0.1% Tween-20 (TBST). After blocking, the membranes were incubated in their respective primary antibodies overnight at 4 °C. The next day, membranes were washed in TBST three times and incubated with secondary antibodies (1:10,000 dilution) for 2 h at room temperature. Blots were washed three times in TBST and then incubated in chemiluminescence ECL reagent and exposed to film. Ponceau staining of total proteins were performed as loading control for each blot and the expression of protein was normalized to its respective loading control. Oxidative stress affects the level of GAPDH or β-actin; therefore, we used ponceau for the blots as a reliable loading control. Densitometry of blots were performed with ImageJ. The antibodies used in the study were p21 (Cat#AB109199, Abcam 1:1000), p53 (Cat# 1:1000, Ab26), phospho-NF-ĸB (Cat#S536, Cell signaling and technology 1:1000), total NF-ĸB (Cat#D14E12, Cell Signaling Technology 1:1000), phospho-Smad3 (Cat#C25A9, Cell signaling and technology 1:1000), total-Smad3 (Cat#C67H9, Cell signaling and technology 1:1000), and Smad4 (Cat#ab40759, Abcam 1:1000). The secondary antibodies used were anti-rabbit (Cat#7074S, Cell Signaling Technology 1:100000) or anti-mouse (Cat#31437, Invitrogen 1:100000).

### Immunofluorescence

Immunofluorescence staining of tissue sections was done as described previously^80^. The paraffin embedded tissues were deparaffinized in xylenes, rehydrated in graded alcohols, followed by antigen retrieval in 0.01 M sodium citrate buffer pH 6.0 and EDTA pH 9.0 with 0.05% Tween 20. Slides were then washed for 5 min in PBS and blocked in 10% goat serum or 10% horse-serum for 1–2 h at room temperature. Next, the slides were incubated overnight in primary antibody at 4 °C. The next day, slides were washed in PBS and incubated in fluorescent secondary goat anti-mouse AlexaFluor 488 (Cat#A11001, Invitrogen 1:1000), 1:1000 or secondary anti-rabbit AlexaFluor 555 (Cat#31460, Invitrogen, 1:1000) for 2 h in the dark at room temperature.

For immunofluorescence staining of cells, obese 3T3-L1 adipocytes were irradiated 3 Gy for 3 consecutive days continuously or 0 Gy. At applicable time points, cells were trypsinzed and cytospun on to glass slides followed by fixation using 4% paraformaldehyde for 15 min. The antibodies used in the present study on the tissue sections and obese 3T3-L1 cells are mouse anti-DNA/RNA Damage (Cat#AB15A3, Abcam 1:400), mouse anti-4-hydroxynoneonal (Cat# BS-6313R, ThermoFisher 1:400) for oxidative stress analysis. To minimize non-specific staining, a Mouse-on-Mouse kit (Vector Laboratories, Newark, CA, USA, FMK-2201) was used according to the manufacturer’s recommendations. To measure immune infiltration, primary rabbit monoclonal anti-CD4 (Cat#11089; Abcam 1:1000), primary rabbit monoclonal anti-F4/80 (Cat# 111101, Abcam 1:50), and primary rabbit monoclonal anti-CD8 (Cat# 217344, Abcam 1:1000) were used. For the senescence and fibrosis experiments, p16 (Cat#51243, Abcam 1:600), p21 (Cat#109199, Abcam 1:500), p53 (Cat#ab26, Abcam 1:400), and α-SMA (Cat#NB300-978, Invitrogen 1:1000) were utilized. The secondary antibodies used were anti-mouse AlexaFluor 488 (Cat#A11001, Invitrogen 1:1000), anti-rabbit 488 (Cat#A-11008, Invitrogen 1:1000), secondary anti-rabbit AlexaFluor 555, (Cat#31460, Invitrogen, 1:1000), or secondary anti-donkey goat AlexaFluor 555 (Cat#A21432, Invitrogen 1:1000). ImageJ software version 1.48 was used to measure the mean fluorescence intensity or the percentage of positive cells per image per mouse. Whole images were analyzed for either the mean fluorescence intensity or percentage of positive cells for all the immunohistochemistry images. DAPI was used to determine the total number of cells per image. All images were captured using a Leica DM4000 B LED microscope (Leica, Deerfield, IL, USA) at 200X. Senescence markers and immune cells infiltration are reported as the percentage of cells per image per mouse.

### β-galactosidase staining

Seven days after radiation, obese 3T3-L1 adipocytes were fixed in 4% paraformaldehyde for 10–15 min. The fixed cells were washed with PBS two times, and incubated with senescence associated beta-galactosidase (SA-β-Gal) staining solution containing 0.1% X-gal, 5 mM potassium ferrocyanide, 5 mM potassium ferricyanide, 150 mM sodium chloride, and 2 mM magnesium chloride in 40 mM citric acid/sodium phosphate solution, pH 6.0 for 48–72 h at 37 °C as described previously^18,49^. An Olympus IX81 inverted microscope was used to capture brightfield images at 200X. Cells showing a green stain (positive-β-Gal stain) were quantified and the data is presented as number of β-Gal^+^ cells per image.

### Statistical analysis

GraphPad Prism v9 was used for all statistical analysis. Mean and standard error of the mean values from three or more independent biological experiments were used for statistical analysis of all in vitro experiments performed. Data were examined for outliers prior to analysis, and none were observed. For changes in oxidative stress, fibrosis, senescence assuming a coefficient of variation of 0.4, alpha = 0.05 to detect a 2.0 change with 85% power, we estimated that we needed 5 mice. Therefore, for in vivo experiments each group consisted of five mice and statistical analyses were performed on the mean values obtained from these five animals per group. A one-way ANOVA followed by post-hoc Tukey’s test for multiple comparisons between multiple groups and unpaired two-tailed Student’s t-test was used to evaluate the differences between two groups. p values < 0.05 were considered statistically significant. Exact p-values are reported and displayed on the graphs within each figure. All graphical representations of data are the mean ± SEM unless otherwise indicated.

## Supplementary Information

Below is the link to the electronic supplementary material.

Supplementary Material 1

## Data Availability

The western blots used in this study have been submitted as a supplementary file in the original uncropped form. Other raw data supporting this study’s findings will be made available from the corresponding author upon reasonable request.
