# Supplementary material for: Irradiated obese adipocytes are prone to oxidative stress, inflammation, senescence, and fibrosis
Source: Sci Rep. 2025 Nov 13;15:39844. doi: 10.1038/s41598-025-23533-7 (PMC12615693; doi:10.1038/s41598-025-23533-7)
Supplement: Supplementary file 1 — Supplementary Material 1 [file 41598_2025_23533_MOESM1_ESM.pdf]

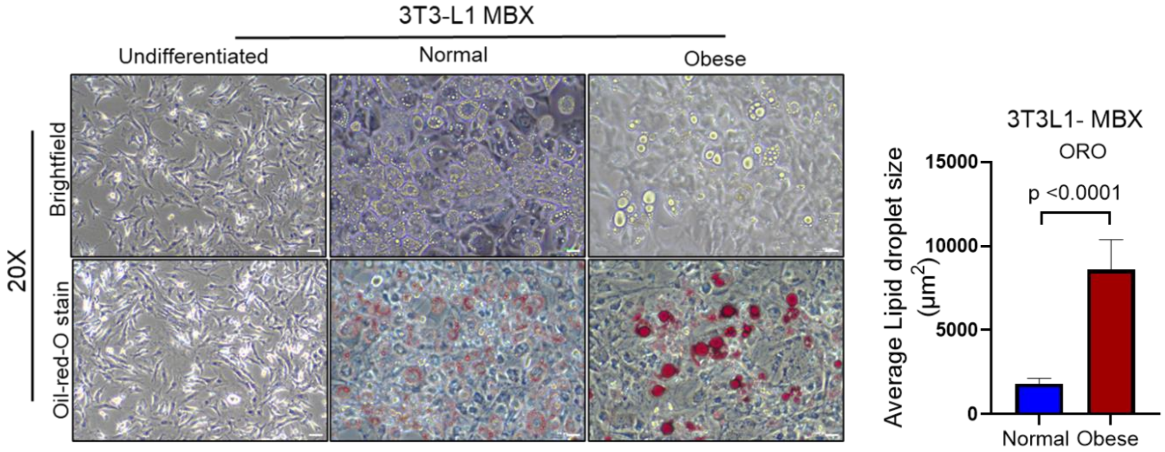

Supplementary Fig. 1 Establishing *in vitro* obesity model. Left, representative brightfield and Oil Red-O stained images. Right, quantification of lipid droplet size of Oil-O-Red images. n=3 biological repeats.

Supplementary Fig.2. Original Blot of Fig.4F

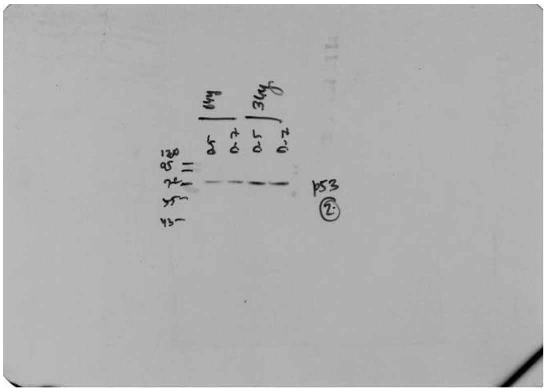

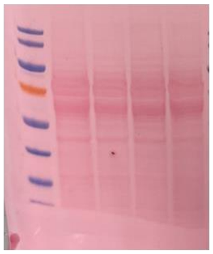

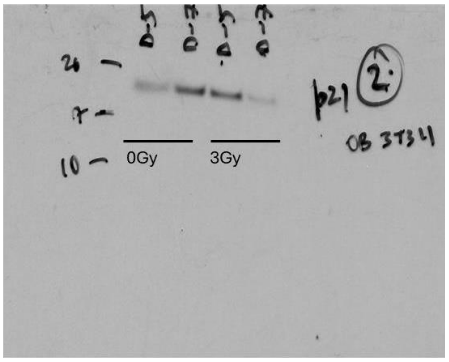

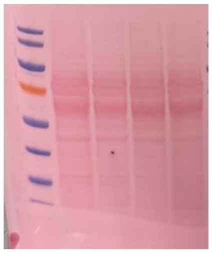

Loading control of p53

Loading control of p21

Supplementary Fig.3 Original blot of Fig.5D

Figure 5D: Original blot

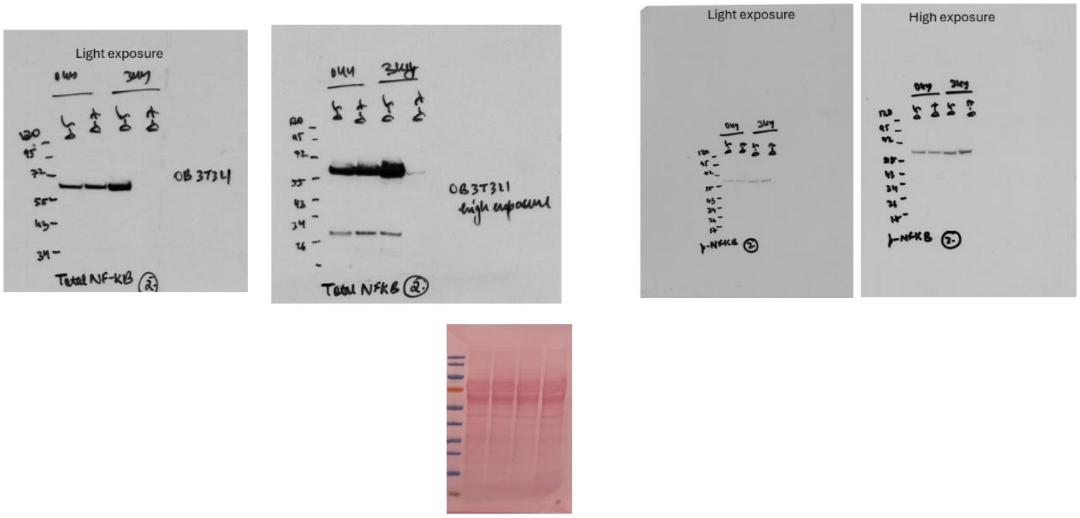

Loading control of phospho and total NF-KB

Supplementary Fig.4 Original blot of Fig.6C

Figure 6C: Original blot

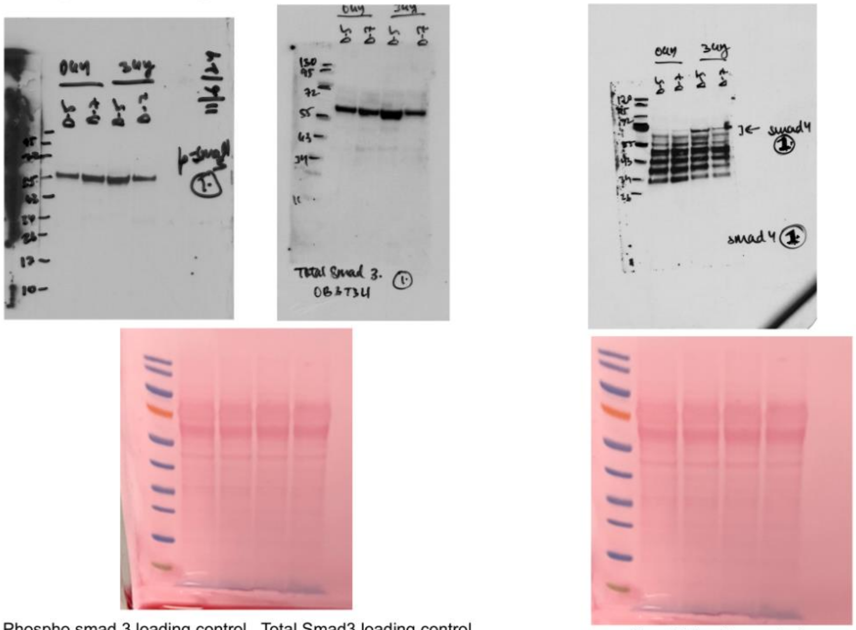

Phospho smad 3 loading control , Total Smad3 loading control

Smad 4 loading control
